# The cancer patient’s perspective of COVID‐19‐induced distress—A cross‐sectional study and a longitudinal comparison of HRQOL assessed before and during the pandemic

**DOI:** 10.1002/cam4.3950

**Published:** 2021-05-10

**Authors:** Karin A. Koinig, Christoph Arnold, Jens Lehmann, Johannes Giesinger, Stefan Köck, Wolfgang Willenbacher, Roman Weger, Bernhard Holzner, Ute Ganswindt, Dominik Wolf, Reinhard Stauder

**Affiliations:** ^1^ Department of Internal Medicine V, Hematology and Oncology Comprehensive Cancer Center Innsbruck (CCCI) Medical University of Innsbruck Innsbruck Austria; ^2^ Department of Radiation Oncology Comprehensive Cancer Center Innsbruck (CCCI) Medical University of Innsbruck Innsbruck Austria; ^3^ University Hospital of Psychiatry II Medical University of Innsbruck Innsbruck Austria; ^4^ OncoTyrol—Center for Personalized Cancer Medicine Innsbruck Austria

**Keywords:** cancer patients, corona virus disease 2019, distress, emotional well‐being, health related quality of life, survey

## Abstract

**Background:**

To permit timely mitigation of adverse effects on overall clinical outcome, it is essential to understand how the pandemic influences distress and health‐related quality of life (HRQOL) in cancer patients during the coronavirus disease 2019 (COVID‐19) pandemic.

**Methods:**

In this cross‐sectional study, adult cancer patients, without COVID‐19 symptoms, completed a 13‐item questionnaire about the pandemic's impacts on distress and everyday‐life; associations with age, sex, or impaired HRQOL were then assessed by binary logistic regressions. In a subsample of patients with HRQOL assessment available from both before and during the pandemic, we evaluated the pandemic's impact on longitudinal changes in HRQOL reported within 6 months before versus during the COVID‐19 lockdown using McNemar's test, and thresholds for clinical importance.

**Results:**

We consecutively enrolled 240 patients with solid (50%) or hematological (50%) cancers. Median age was 67 years, 46% were females. The majority ranked heeding their health (80%) and keeping their appointment schedule in hospital (78%) as important. Being younger than 60, or aged 60–70 was independently associated with limitations in everyday life (OR = 3.57, *p* < 0.001; and 2.05, *p* = 0.038); female individuals and those with restricted emotional functioning were more distressed by the COVID‐19 situation (OR = 2.47, *p* = 0.040; and 3.17, *p* = 0.019); the latter group was also significantly more concerned about being a patient at risk (OR = 2.21, *p* = 0.029). Interestingly, in a subsample of patients (*n* = 47), longitudinal comparisons pre‐ versus during the pandemic revealed that HRQOL was not substantially affected by the pandemic.

**Conclusion:**

Particularly younger and female cancer patients, and those with impaired emotional functioning are distressed by COVID‐19. During the first COVID‐19 lockdown, cancer patients remained predominantly resilient. This analysis highlights the need to mitigate distress situations in vulnerable patients and thereby enhance resilience during pandemics.

## Graphical abstract

In this cross‐sectional study of 240 cancer patients, the majority reported heeding their own health and wished to maintain their appointment schedule. We observed significantly more restrictions in younger and female patients. Comparing quality of life in the timespan before versus during the COVID‐19 pandemic revealed that quality of life was not substantially affected by the COVID‐19 crisis but indicated that cancer patients generally cope unexpectedly well with the pandemic, being already used to restrictions in their everyday life.

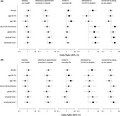

## 1 INTRODUCTION

The exponential spread of the severe acute respiratory syndrome coronavirus‐2 (SARS‐CoV‐2) across countries prompted unprecedented and exceptional restrictions in everyday life. As a consequence, the COVID‐19 pandemic affects the mental health of the general population,^1^, ^2^ of medical staff,^3^, ^4^, ^5^ and of those infected with SARS‐CoV‐2.^6^ Only very little information is available for the most vulnerable populations, such as cancer patients.^7^, ^8^, ^9^, ^10^ As maintaining quality of life (HRQOL) remains one of the treatment goals in cancer care,^11^, ^12^ there is an urgent need to address how the pandemic affects mental health from the patient's perspective. This knowledge is a prerequisite for adequate psychosocial management in mitigating distress, thereby improving emotional well‐being in a situation that confronts individuals with two potentially life‐threatening conditions, that is, cancer and a viral pandemic.

When the number of COVID‐19 cases started to rise across Europe in February 2020, the region became one of the early hotspots, with COVID‐19 infections spreading from ski resorts. The local government initiated strict quarantine measures starting mid‐March including a stay‐at‐home order; a ban on travel between counties; homeschooling; a ban on sports, and the closing of all skiing areas. As case numbers kept increasing, wearing face masks in shops and public places became compulsory in late March. Following a drop in the number of new infections, the government step‐by‐step eased restrictions by mid‐June until the second wave ramped up in late September.

Our research objectives were to study cancer patients’ perception of the COVID‐19 pandemic and its impact on their everyday life during the lockdown. In order to gain a better understanding of the factors influencing patients’ distress, we assessed whether the situation was experienced differently depending on age, sex, or impairments reported in health‐related quality of life (HRQOL). In addition, we aimed to capture changes in self‐reported HRQOL during the very restrictive phase of COVID‐19 lockdown versus 6 months prior to the first regional incidence and spread of COVID‐19.

## 2 METHODS

### 2.1 Study design and participants

In this single‐center, cross‐sectional study we consecutively enrolled adult cancer patients. The survey was launched on 20 April 2020, during the region's most restrictive COVID‐19 lockdown measures, and was terminated on 18 June 2020, 3 days after major loosening of restrictions. In addition, in a subsample of patients, longitudinal data on HRQOL were available for comparison from before the onset of the pandemic. Patients were eligible if they were diagnosed with a solid tumor or a hematological malignancy, were aged 18 or older, had neither symptoms nor any clinical or laboratory evidence of a COVID‐19 infection and had signed the written informed consent.

A team of clinical psychologists compiled a 13‐item survey on COVID‐19’s impact on cancer patient's distress and everyday life (see Supporting information S1‐S6). The survey assessed the patients’ worries and stress caused by the COVID‐19 situation, their satisfaction with government efforts and information. It also captured whether a patient's hospital appointments had been postponed. Patients with ongoing radiation therapy additionally reported any problems with their transport service. For each question, participants selected one option from “very much,” “quite a bit,” “a little,” and “not at all.” In four of the survey questions, patients had the option to additionally specify (i) the kind of distress they had experienced, (ii) the type of limitations on their everyday life, (iii) how they had heeded their health, and (iv) what additional information they would have wanted. The questionnaires were available either as printed forms, or in electronic form with the option to answer remotely via the Computer‐based Health Evaluation System (CHES).^13^

In addition, patients completed the European Organisation for Research and Treatment of Cancer Quality of Life Questionnaire (EORTC QLQ‐C30) that uses 30 questions to assesses five functional scales (physical, role, emotional, cognitive, and social), nine symptom scales (fatigue, nausea and vomiting, pain, dyspnea, insomnia, appetite loss, constipation, diarrhea, and financial difficulties), a global health status/QoL scale (global QOL), and the EORTC‐QLQ‐C30 summary score (QLQ‐C30‐SumScore).^14^ A high score in functioning is favorable, while a high score in symptoms is unfavorable. To address restrictions in relation to the COVID‐19 situation we focused on QLQ‐C30‐SumScore, global QOL, and the scales for physical, emotional, social and role functioning; while we excluded the symptom scales which are more strongly affected by the type and status of the underlying disease itself. We used established thresholds of clinical importance^15^ to identify patients with impaired functioning. Moreover, national general population normative data^16^ were used to discriminate against patients restricted in global QOL or EORTC‐QLQ‐C30 summary score, as thresholds for these two scales are not available.

Within a subsample of the study cohort, we evaluated longitudinal changes in HRQOL in patients who had additionally completed an EORTC QLQ‐C30 assessment via CHES in the CLL and the national Myeloma registries^17^, ^18^ less than 6 months prior to first incidence of COVID‐19 in the country (pre‐Covid‐19, i.e., before 29 February 2020, patients with multiple myeloma and chronic lymphocytic leukemia). The focus of longitudinal analyses was on emotional, social, role, physical functions, global QOL, and QLQ‐C30‐SumScore. We distinguished clinically meaningful changes following Cocks et al.^19^

### 2.2 Outcomes

The primary endpoint was to assess the degree of the impact of the COVID‐19 pandemic on cancer patients’ distress and everyday life. Secondary endpoints were (1) whether the risk of experiencing the COVID‐19 pandemic as having an impact on everyday life was significantly related to age, sex, or clinically relevant impairments in self‐reported HRQOL, with a focus on global QOL, QLQ‐C30‐SumScore, emotional, social, role, or physical functioning; and (2) whether patients reported clinically meaningful changes in HRQOL during the most restrictive lockdown phase as compared to the time before COVID‐19 became incident in the region.

### 2.3 Statistical analyses

We used descriptive statistics to show the impact of COVID‐19 on the everyday life of cancer patients, reporting categorical data as frequencies and quantitative data as means with standard deviations and inter‐quartile ranges (IQR). We dichotomized the answers “very” and “quite a bit” versus “a little,” and “not at all” in the data evaluation. We stratified the patients’ answers by age, sex, cancer type (solid vs. hematological), and HRQOL (global QOL, QLQ‐C30‐SumScore, emotional, social, role, or physical functioning). To explore risk factors or their interactions, associated with a high impact of COVID‐19 on cancer patients’ everyday life, we used univariable and multivariable logistic regression models to estimate odds ratios (OR) and 95% confidence limits (Cl), testing the variables age, sex, and impaired HRQOL. A two‐sided *p* < 0.05 was considered statistically significant. We evaluated the overall models with 2 log‐likelihood, goodness of fit, and the models’ effectiveness with Nagelkerke R2. The chi‐squared test was used when comparing unpaired samples. We evaluated the significance of changes in longitudinal HRQOL with McNemar's test. All analyses were performed using SPSS (IBM SPSS statistics version 24).

## 3 RESULTS

### 3.1 Participants

We consecutively enrolled 240 patients with cancer: 120 (50%) suffered from a solid tumor and 120 (50%) from a hematological malignancy. The most common solid tumor types were breast (19%), prostate (9%), and lung cancer (5%); the most common hematological malignancies were chronic lymphocytic leukemia (17%), multiple myeloma (16%), and myelodysplastic syndromes (10%). Mean age was 67 years (SD 12.5), and 46% were female (Table 1). All solid cancer patients were evaluated during tumor‐specific therapy in the hospital, while patients with hematological malignancies had either follow‐up visits or a planned treatment in the hospital, or answered remotely via the patient portal (*n* = 37). Longitudinal data on HRQOL were available in a subsample of 47 patients of the cross‐sectional cohort.

**TABLE 1:** Characteristics of the total cohort in subgroups as defined by tumor type

Characteristic	All (*n* = 240)	Solid (*n* = 120)	Hematologic (*n* = 120)	Longitudinal^c^ (*n* = 47)
Sex, *n* (%)				
Male	129 (54)	60 (50)	69 (58)	31 (66)
Female	111 (46)	60 (50)	51 (43)	16 (34)
Age, mean (SD)	67 (13)	64 (14)	70 (104)	67 (9)
Age groups, *n* (%)				
<40	6 (3)	6 (5)	0 (0)	0 (0)
40 to <50	15 (6)	12 (10)	3 (3)	1 (2)
50 to <60	49 (20)	29 (24)	20 (17)	11 (24)
60 to <70	66 (28)	29 (24)	37 (31)	18 (38)
70 to <80	72 (30)	34 (28)	38 (32)	14 (30)
80 to <90	30 (13)	9 (8)	21 (18)	3 (6)
>=90	2 (1)	1 (1)	1 (1)	0 (0)
Entities, *n* (%)				
Breast cancer	45 (19)			
Chronic lymphocytic leukemia (CLL)	40 (17)			21 (45)
Multiple myeloma (MM)	38 (16)			26 (55)
Myelodysplastic syndromes (MDS)	24 (10)			
Prostate cancer	22 (9)			
Lung cancer	12 (5)			
Squamous cell carcinoma of head and neck (HNSCC)	12 (5)			
Acute myeloid leukemia (AML)	8 (3)			
Non‐Hodgkin's lymphoma (NHL)	7 (3)			
Other^a^	32 (13)			
Risk category, *n* (%)^b^				
High‐risk	80 (33)	46 (38)	34 (28)	11 (23)
Low‐risk	160 (67)	74 (62)	86 (72)	36 (77)
Treatment schedule, *n* (%)				
As planned	199 (83)	99 (83)	100 (83)	39 (83)

^a^ Information about subtypes occurring in less than 2% (*n* < 5) of patients (see supporting information S1‐S6).

^b^ Risk classification (see supporting information S1‐S6).

^c^ The longitudinal cohort is a subsample of the cross‐sectional study of patients with HRQOL assessment available form before the onset of the pandemic

### 3.2 Perception of the COVID‐19 pandemic's impact on distress and everyday life

**FIGURE 1:**
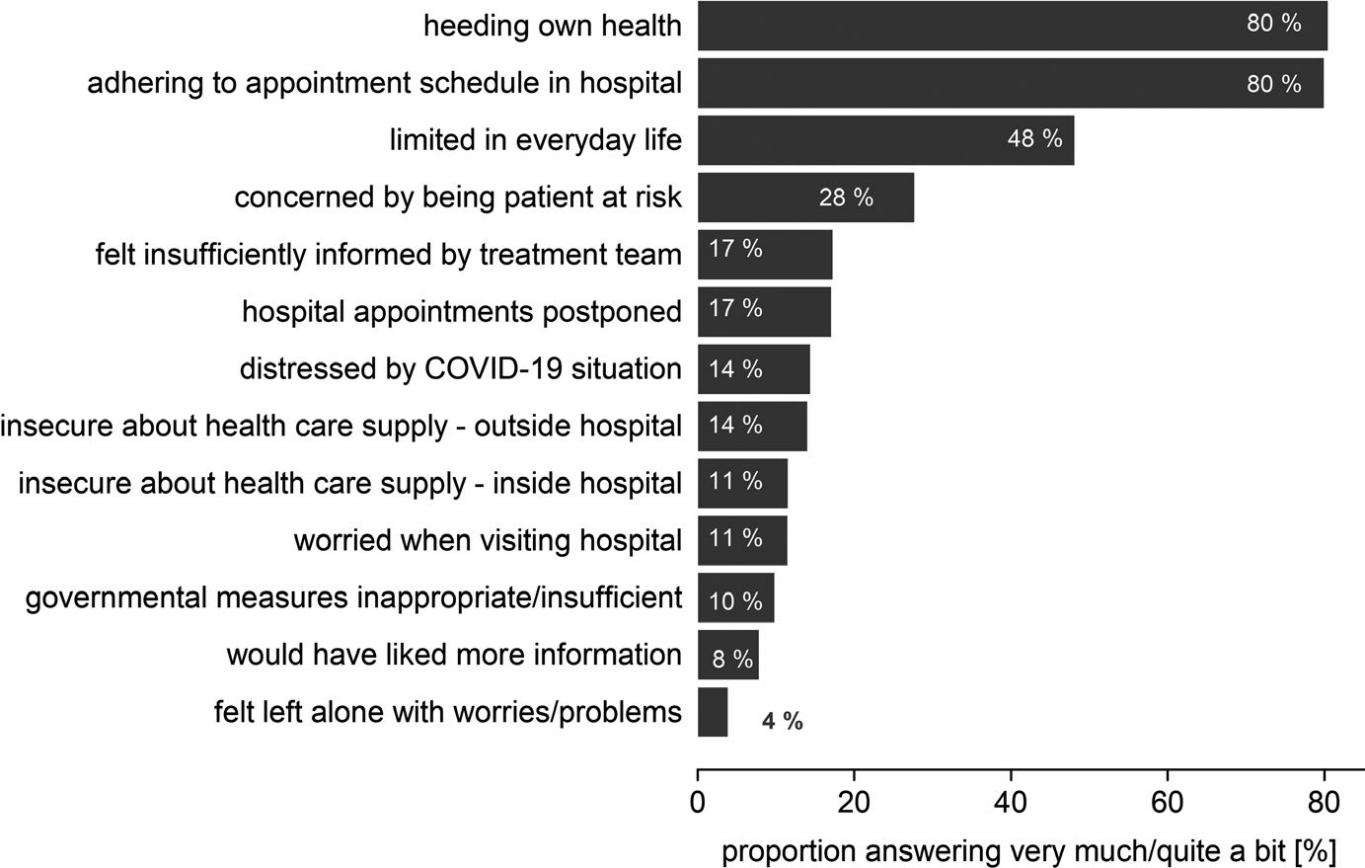
Answers to the survey of COVID‐19’s impact on cancer patients’ distress and everyday life. Proportion of patients answering “very much” or “quite a bit” in the survey

We observed the patients’ perception of the COVID‐19 pandemic's impact on distress and everyday life (Figure 1). The vast majority of the patients (80%) stated that they were heeding their own health during the COVID‐19 pandemic, and emphasized the importance of adhering to their hospital appointment schedule. In fact, only 17% reported having postponed their hospital appointments. Overall, only few patients were worried about visiting the hospital (11%), either with regard to access to medical care inside (11%) or outside the hospital (14%).

Daily routine was limited for 48% of the patients. Around 40% specified these limitations, mentioning, for example, job loss; problems with organizing childcare; a sense of isolation; missing personal contacts, especially with their children, grandchildren, and friends; or feeling imprisoned. Still, only 4% felt they were left alone with their problems and worries.

Despite belonging to a high‐risk group for COVID‐19, only a minority was worried about being an at‐risk patient (28%), or felt distressed by the COVID‐19 pandemic (14%). The majority of patients felt sufficiently informed by their treatment team (83%). However, of the patients who had answered remotely *via* the virtual patient portal, without direct contact to their physician, 49% felt insufficiently informed by their treatment team (*p* < 0.001), and 17% would have liked more information (*p* < 0.001, supporting Figure S1‐S6) as compared to the patients who answered the questionnaires during a visit to the out‐patient clinic. The majority of patients considered the government rules to be justified (90%).

### 3.3 Association of patients’ responses with age, sex, or impairments in HRQOL

**FIGURE 2:**
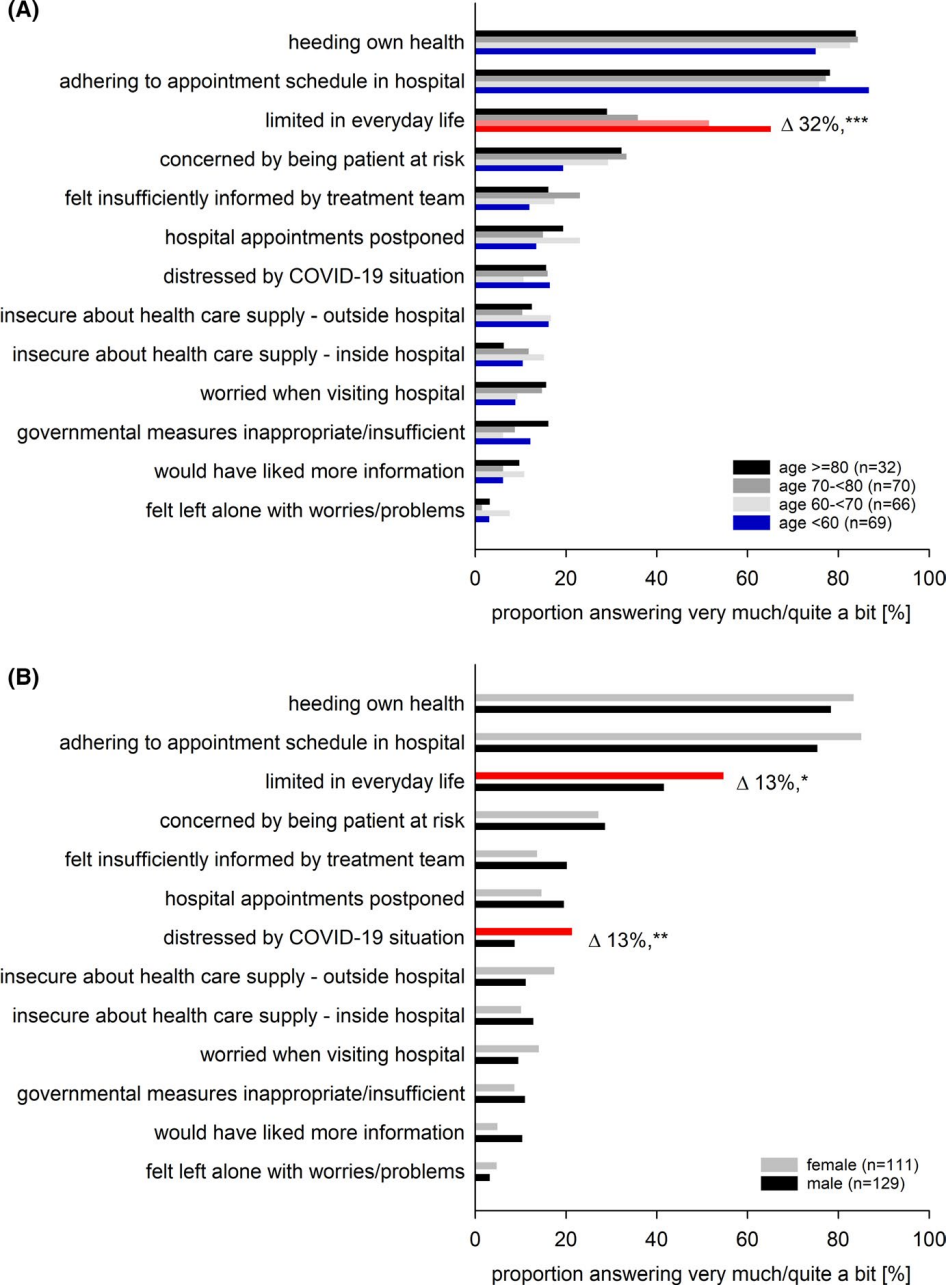
Stratification by age and sex of the patients’ answers in the survey of COVID‐19’s impact on cancer patients’ distress and everyday life. Proportion of patients answering “very much” or “quite a bit” stratified by (A) age subgroups and (B) sex. Note that patients younger than 60 years and those aged 60–70 years reported significantly greater limitations in everyday life (*p* < 0.001, and *p* 0.021), while we observed no differences by age in the responses to any other question. Female patients reported significantly greater limitations in everyday life (*p* = 0.047) and were significantly more strongly distressed by the COVID‐19 situation (*p* 0.008). In contrast, the responses to all other questions did not differ significantly between females and males. Red: significant differences in univariable linear regression (for odds ratios, see Figure 3A). Δ differences between categories, ***p < 0.001, **p < 0.001, *p < 0.01

**FIGURE 3:**
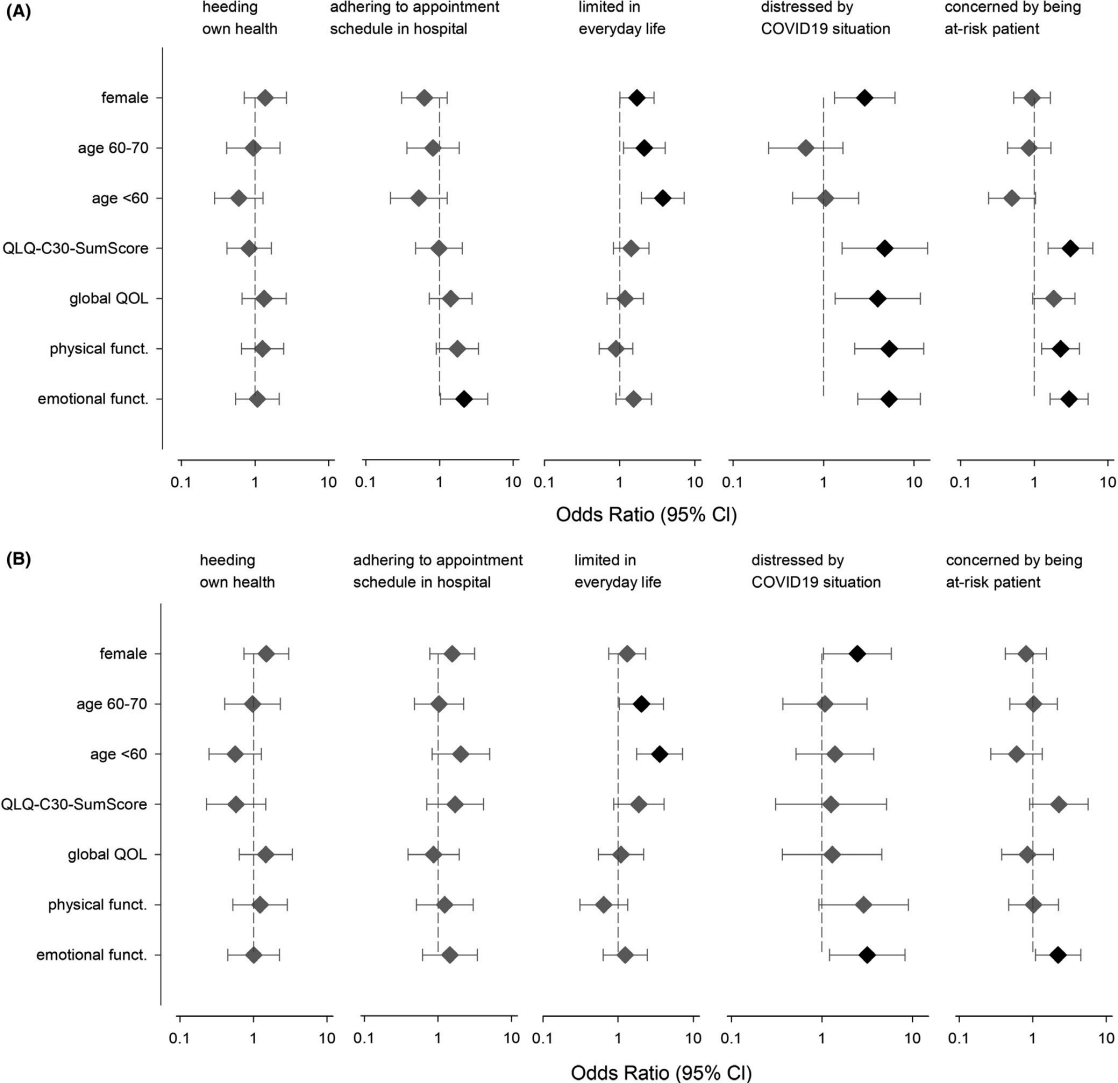
Association between sex, age, and HRQOL on five items of the survey of COVID‐19’s impact on cancer patients’ distress and everyday life. Depiction as odds ratios with 95% confidence intervals. Significant associations are depicted in black, not significant ones in gray. (A) Univariable model and (B) multivariable model adjusted for age, sex, and HRQOL. Females compared to males. Age subgroups compared to individuals older than 70. EORTC‐QLQ‐C30 dimensions (summary score, global QOL, and physical and emotional functioning) compared between individuals with and without impairments. Additional details see supporting Table S1‐S6

We assessed whether the patients’ responses were associated with age, sex, or impairments in HRQOL (Figures 2 and 3, supporting Table S1‐S6). Patients with impaired emotional functioning significantly more frequently wished to adhere to their appointment schedule (univariable analyses: OR = 2.15, 95% CI = 1.03–4.50, *p* = 0.041). Moreover, limitations in everyday life were significantly more frequent in female patients (OR = 1.70, 1.01–2.86, *p* = 0.047), in patients younger than 60 years (OR = 3.74, 1.94–7.24, *p* < 0.001), and in patients aged 60–70 (OR = 2.12, 1.12–4.02, *p* = 0.021). Females (OR = 2.85, 1.32–6.17, *p* = 0.008) and those individuals with restrictions in HRQOL reported being more distressed by the COVID‐19 pandemic (QLQ‐C30‐SumScore: OR = 4.75, 1.60–14.11, *p* = 0.005; global QOL: OR = 3.98, 1.35–11.76, *p* = 0.012; physical functioning OR = 5.33, 2.22–12.81, *p* < 0.001; emotional functioning: OR = 5.30, 2.39–11.76, *p* < 0.001). A pronounced concern about being a patient at risk was associated with restrictions in the QLQ‐C30‐SumScore (OR = 3.10, 1.53–6.26, *p* = 0.002), physical (OR = 32.27, 1.26–4.09, *p* = 0.006), and emotional functioning (OR = 2.96, 1.64–5.36, *p* < 0.001).

In the multivariable analyses, adjusted for age, sex, QLQ‐C30‐SumScore, global QOL, physical, and emotional functioning, the following associations remained significant: (a) limitations in everyday life with being younger than 60 (OR = 3.57, 1.77–7.19, *p* < 0.001), or aged 60–70 (OR = 2.05, 1.04–4.02, *p* = 0.038); (b) being distressed by the COVID‐19 situation with being female (OR = 2.47, 1.04–5.86, *p* = 0.040) or restricted in emotional functioning (OR = 3.17, 1.21–8.27, *p* = 0.019); (c) being more concerned about being a patient at risk with being restricted in emotional functioning (OR = 2.21, 1.09–4.49, *p* = 0.029) (Figure 3B, supporting Table  S1‐S6).

### 3.4 Intraindividual, longitudinal changes in HRQOL

We assessed intraindividual, longitudinal changes in HRQOL caused by the COVID‐19 pandemic. Longitudinal HRQOL was available for 47 of the participants for the two timepoints, namely prior to the onset of the COVID‐19 pandemic versus during the COVID‐19 lockdown. These patients had a similar age distribution (mean 66.7 years, SD 9.2), but a smaller proportion of females (34%) as compared to the rest of the cohort. Interestingly, the COVID‐19 pandemic only marginally affected emotional functioning (Δ = −2.30 score points), physical functioning (Δ = 1.84), role functioning (Δ = 0.35), social functioning (Δ = 2.13), and global QOL (Δ = −1.95; supporting Figure S1‐S6), values that were all below the threshold of clinically meaningful differences.^8^ Only very few individual patients reported strong changes (Δ>20),^20^ which included either improvement or deterioration of physical, role, emotional, or social functioning, or of global QOL (supporting Figure S1‐S6).

**FIGURE 4:**
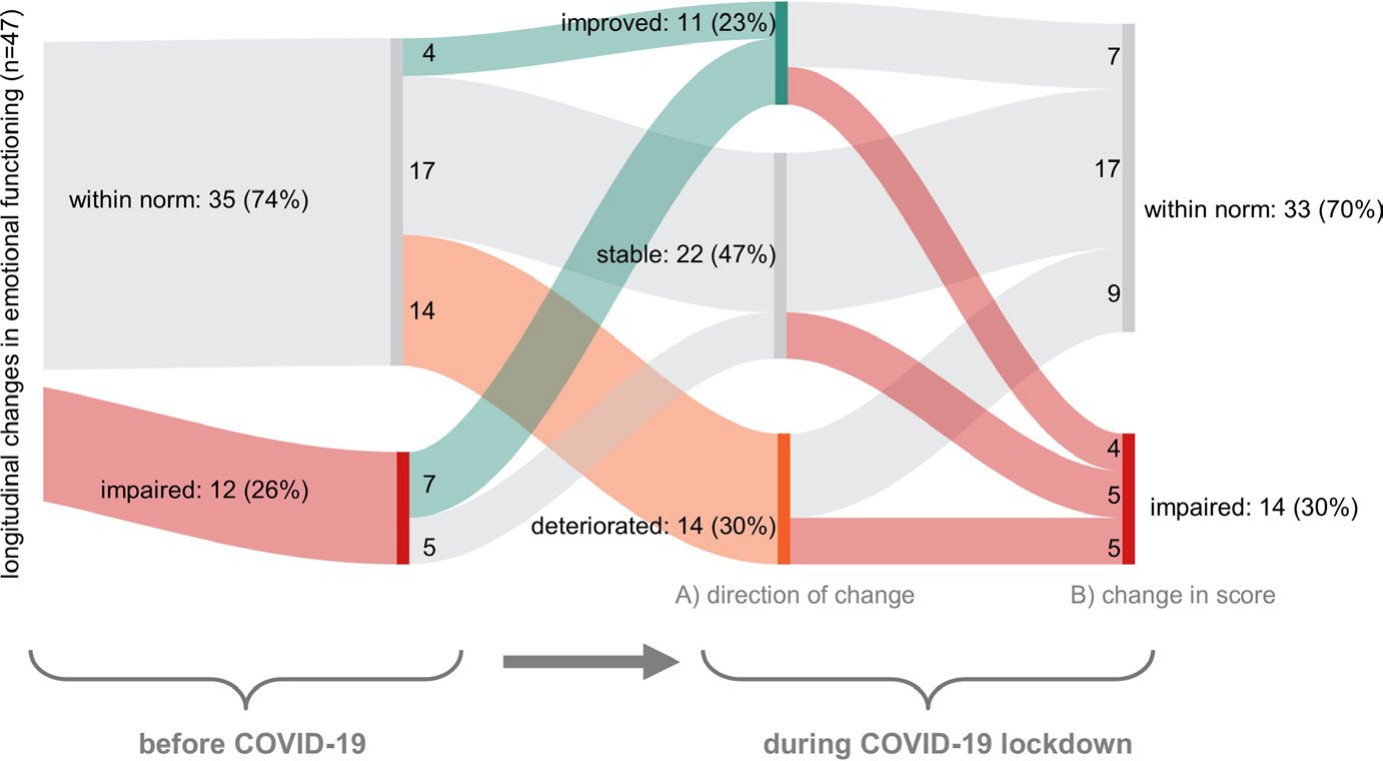
Longitudinal changes in emotional functioning assessed in 47 patients before and during the COVID‐19 lockdown. Scores during lockdown are classified in two ways: (A) improvement versus stable versus deterioration in score, and (B) scoring as within norm versus as impaired. Note that all deteriorations (*n* = 14) occurred in patients that had not reported restrictions in their assessment prior to the COVID‐19 lockdown. Of these 14 patients, five became impaired, while nine remained within the norm. In contrast, of the twelve patients who had reported impaired emotional functioning before the COVID‐19 lockdown, none deteriorated, while seven improved (but four remained impaired), and five remained stable. A large proportion of patients remained stable (*n* = 22, 46%). Red: impaired. Gray: not impaired or stable. Green: improved. Orange: deteriorated

As emotional functioning was associated with a higher risk of being distressed and of being concerned about being a patient at risk, we further explored which of the 47 patients reported longitudinal changes in emotional functioning (Figure 4). While we observed a clinically relevant improvement or deterioration in a small number of patients (*n* = 7 and *n* = 5, respectively), these changes cannot be separated from a regression to the mean effect. On average, patients reporting a deterioration were slightly younger (64.7 years) than were those who remained stable (68.0) or who improved (66.7), independent of sex. However, overall changes in the score were independent of age (*R* ^2^ = 0.02).

## 4 DISCUSSION

Patient‐partnered clinical research in oncology is key to understanding the impact of the pandemic on HRQOL in patients with malignancies. Our study highlights the impact of the pandemic on cancer patients’ distress, and in particular we were able to compare these data intraindividually with HRQOL parameters documented in the pre‐COVID‐19 era. Contrary to our expectations, only a minority of the patients felt distressed by the COVID‐19 situation itself, despite the higher risk for an adverse outcome if infected with SARS‐CoV‐2. It was, however, key for cancer patients that their cancer care remained unaffected, reflected by their wish to maintain their appointment schedule. Particularly female, or emotionally impaired individuals, or those of young age felt distressed by the pandemic. The latter felt more limited by the lockdown in their everyday life. Notably, intraindividual longitudinal analyses of HRQOL (before vs. during the COVID‐19 lockdown) demonstrated a minor impact of COVID‐19 on emotional and physical functioning, global QOL and the QLQ‐C30‐SumScore. These data highlight the fact that cancer patients coped unexpectedly well with distress caused by the pandemic.

Still, our study indicates a greater vulnerability of younger and female cancer patients, suggesting that these patients might require more vigilance and special counselling to improve their resilience to the pandemic. Age and sex have been reported to play a role in coping with the COVID‐19 pandemic, both in the general population^21^, ^22^, ^23^ and in cancer patients. Fewer restrictions in emotional functioning and global QOL score were associated with increased age, and male gender in patients with cancer,^24^ while females or those younger than 65 years reported greater distress, or anxiety and depression.^25^, ^26^ This shows that younger individuals in general feel more restricted by stay‐at‐home orders.^2^, ^27^, ^28^

As recent reports suggested an alarming increase in distress in cancer patients during the COVID‐19 pandemic,^29^ we were surprised that only 28% of our study patients felt distressed by the pandemic. At the same time, our patients reported being in strong agreement with government precautionary measures (90%), which might have mitigated the distress by permitting them to feel adequately protected. Overall, the cancer patients in this study seemed to be well aware of the importance of heeding their own health and reported high compliance with social distancing measures. Similarly, German cancer patients showed no elevated level of distress, anxiety, or COVID‐19‐related fear.^30^ In China, the decrease in psychological impact (despite a concurrent strong regional increase in COVID‐19 cases) had been attributed to the individual's satisfaction with appropriate health information and personal precautionary measures.^31^ Intercountry differences in distress might thus be related to lower incidence rates and fewer controversies about timely political measures. However, the current second lockdown (starting in November 2020) might be perceived differently.

In order to better gauge the COVID‐19‐pandemic's specific impact on distress and HRQOL, we studied longitudinal changes intraindividually pre‐COVID‐19 versus during the first lockdown. This concept stands in contrast to other currently available studies of longitudinal changes in HRQOL in cancer patients. While we follow intraindividual changes before and during the COVID‐19 pandemic, these studies referred to reference cohorts.^24^, ^32^, ^33^ These studies reported contrasting trends in HRQOL and either observed no clinically significant differences in global QOL^24^, ^33^ and emotional functioning^24^ ; or reported clinically meaningful decreases in global QOL, cognitive and social functioning, but not in emotional or physical functioning.^32^ In comparison, in our study of intraindividual change in HRQOL, we did not observe a significant longitudinal deterioration in HRQOL. Likewise, in a conceptually similar intraindividual study, patients with multiples sclerosis reported no increase in anxiety and depression as compared to their routine neurophysiological and QOL evaluation within 6 months preceding the COVID‐19 lockdown.^34^ In fact, these patients even reported better social functioning and sexual satisfaction.

Patients who had already been accustomed to restrictions in their everyday life seem to cope better with the additional restrictions imposed by the COVID‐19 pandemic. For example, patients with cancer might already be more used to social distancing to protect themselves from infections, so that the lockdown did not additionally affect them as strongly in role and functioning. Ultimately, as their life had already been destabilized by the cancer diagnosis, these individuals already deal well with a potentially life‐threatening situation. This may in part explain the finding that in most patients the pandemic did not substantially aggravate distress. At the same time, the lockdown increases the social proximity to family members, especially those living in the same household, thus potentially further alleviating emotional distress. In fact, loneliness was hardly reported in our study. In addition, the availability of more time for staying physically active might also serve to mitigate impacts on emotional well‐being during lockdowns.^35^, ^36^ Overall, this indicates greater psychological resilience on the part of cancer patients despite their heightened vulnerability when infected with SARS‐CoV‐2, suggesting something like a pandemic win for patients who were already restricted before the pandemic and are now facing a situation more similar to that of the general population.

### 4.1 Limitations

Given that most patients (85%) were questioned during their hospital visit, the proportion of patients whose appointments had been postponed (17%) might be underestimated, especially the proportion of patients who had no urgent need to visit the hospital for an ongoing treatment or due to a deterioration in their health status. Additionally, longitudinal data were available only for a limited subgroup of patients (*n* = 47). While this is a relatively small number, these intraindividual data capture changes more precisely than does a comparison with general population norms. Moreover, this permits us to single out patients with particularly strong changes or resilience. Furthermore, the cohort encompasses a heterogeneous set of cancer patients. However, when comparing the responses given by patients with solid tumors and those with hematological malignancies and the responses given by the total cohort, we did not observe significant differences. Overall, the impact of the COVID‐19 pandemic will vary over time. We surveyed cancer patients during the first surge of the pandemic. The current increase in numbers and a prolongation of measures might be perceived differently.

## 5 CONCLUSION

The data presented here emphasize the need to capture limitations and distress caused by the COVID‐19 pandemic, particularly in younger and in female patients. Early identification of restrictions might mitigate the impact on everyday life caused by the COVID‐19 pandemic by individualizing interventions that assist in coping with distress and stabilizing emotional well‐being during the COVID‐19 pandemic. Obviously, the basic ability of cancer patients to cope with the pandemic is already high. However, patients clearly indicated that maintaining their appointment schedule is very important, which in addition to the disease‐focused aspect of avoiding delays in essential diagnostics, check‐ups, and/or therapies also supports that cancer care concepts must be maintained in order to reduce additional distress for cancer patients.

## CONFLICT OF INTEREST

Outside this study, DW reports having received personal fees from Roche, Pfizer, Gilead, Novartis, Abbvie, BMs, Celgene, and Amgen; RS reports research funding and honoraria from Celgene, Teva, and Novartis; WW reports funding or personal fees from Abbvie, AMGEN, BMS‐Celgene, Fujimoto, Gilead, GSK, Incyte, Janssen, Novartis, Merck, MorphoSys, Pfizer, Roche, Sandoz, Sanofi, and Takeda; UG reports personal fees from Bayer Vital, Takeda, MERCK, Merck Serono, Roche, BMS, and MSD. All remaining authors declare no conflicts of interests.

## ETHICS STATEMENT

The study was approved by the Ethics Committee of the Medical University of Innsbruck (1264/2020) and is registered on ClincialTrials.gov (NCT04649320).

## Supporting information

Supplementary Material

## Data Availability

The data that support the findings of this study are available from the corresponding author upon reasonable request.
