# Supplementary material for: The cancer patient’s perspective of COVID‐19‐induced distress—A cross‐sectional study and a longitudinal comparison of HRQOL assessed before and during the pandemic
Source: Cancer Med. 2021 May 10;10(12):3928–37. doi: 10.1002/cam4.3950 (PMC8209623; doi:10.1002/cam4.3950)
Supplement: Supplementary file 1 — Supplementary Material [file CAM4-10--s001.pdf]

# Supporting Information S1:

**Supporting Information S1: Characteristics of the total cohort, and in subgroups as defined by the tumour type. See Table 1.**

**Additional information about cancer subtypes occurring in less than 2 % (n <5) of the patients:**

† In 32 patients a cancer with a frequency of less than 2% was diagnosed: Glioma 1.7 %, Melanoma 1.7 %, Rectal cancer 1.7 %, Anal cancer 1.25 %, Bladder cancer 1.25%, Cervical cancer 1.25%, Myeloproliferative neoplasms 0.84%, Squamous cell skin cancer 0.84%, Sarcoma 0.84%, Chronic myelomonocytic leukaemia 0.42%, Carcinoma of unknown primary (CUP) 0.42%, Merkel cell carcinoma 0.42%, Oesophageal cancer 0.42%, Vulvar cancer 0.42%.

**Risk classification:**

‡ Risk was classified as favourable/low in the following subgroups: MM ISS stage 1 or 2, follicular NHL grade 1 or 2, CLL/SLL TP53 Wildtype or IgHV mutated, MDS IPSSR very low/low/intermediate and all cases of HCL, M. Waldenström or marginal cell lymphoma. Unfavourable subtypes included MM ISS stage 3, follicular NHL grade 3, CLL/SLL TP53 mutated or IgHv Wildtype, MDS IPSSR high/very high and all cases of DLBCL, Burkitt-NHL, T-NHL, AML, and ALL. In MPN, CMML and in Mantle Cell NHL classification was based on the disease course and the therapy response. In patients receiving radiation therapy the following subtypes were classified as low risk: breast cancer patients receiving adjuvant after breast-preserving therapy, primary prostate cancer patients. All patients with metastases receiving palliative radiation therapy were classified as unfavourable/high risk. All other patients with radiation therapy were graded according to disease course and tumour stage.

**NOTE:**

Considering that the study was designed to cover cancer patients in general and thus to include heterogeneous cancer types, we restricted our comparison to solid versus hematological cancer, but intentionally did not stratify our analyses further by cancer subtypes, stages or treatments

# Supporting Information S2: questionnaire

## COVID-19 specific questions

Dear patient,

In order for us, as your treatment team at the University Hospital , to better understand the impact of the situation regarding COVID-19 (so-called "coronavirus") on your everyday life, we ask you to answer the following short questions

1. Has one of your outpatient or CCCI appointments (e.g. a follow-up or control appointment) been postponed during the COVID-19 pandemic? ◯ Yes ◯ No

| | Not at all | A little | Quite a bit | Very much |
|---|---|---|---|---|
| 2. Are you currently worried when you are visiting the hospital for an appointment? | ① | ② | ③ | ④ |
| 3. Is it important to you that your usual frequency of visits is maintained? | ① | ② | ③ | ④ |
| 4. Are you distressed by the current situation regarding COVID-19? **IF YES**, what kind of distress do you experience? | ① | ② | ③ | ④ |
| 5. Are you concerned about being a risk patient for COVID-19 as a cancer patient? | ① | ② | ③ | ④ |
| 6. In the past few weeks: Did you feel insecure whether access to medical care at the hospital can be provided? | ① | ② | ③ | ④ |
| 7. In the past few weeks: Did you feel insecure whether access to medical care **outside** the hospital can be provided? | ① | ② | ③ | ④ |
| 8. In the past few weeks: Did you felt left alone with the current problems and worries? | ① | ② | ③ | ④ |

9. Has the COVID-19 pandemic led to limitations in your everyday life? ◯ Yes ◯ No

   **IF YES,** what kind of limitations? ______________________

| | Not at all | A little | Quite a bit | Very much |
|---|---|---|---|---|
| 10. Are you heeding your own health during the COVID-19 pandemic? | ① | ② | ③ | ④ |
| **IF YES,** how are you currently heeding your own health? ______________________ | | | | |
| 11. Do you consider the governmental measures to protect risk groups to be appropriate/sufficient? | ① | ② | ③ | ④ |
| 12. Do you feel that your treatment team at the hospital has provided you with sufficient information on how to deal with the COVID-19 situation? | ① | ② | ③ | ④ |

13. Would you have liked more information? ◯ Yes ◯ No

    **IF YES,** what kind of information? ______________________

Additional question for patients with radiation therapy

| | | | | |
|---|---|---|---|---|
| 14. Did you experience problems with your transport service to the daily radiation therapy? | ① | ② | ③ | ④ |

# Supporting Figure S3

**Supporting Figure S3: Comparison of answers to the COVID-19 survey by the total cohort *versus* patients with solid tumours *versus* by patients with haematological malignancies**

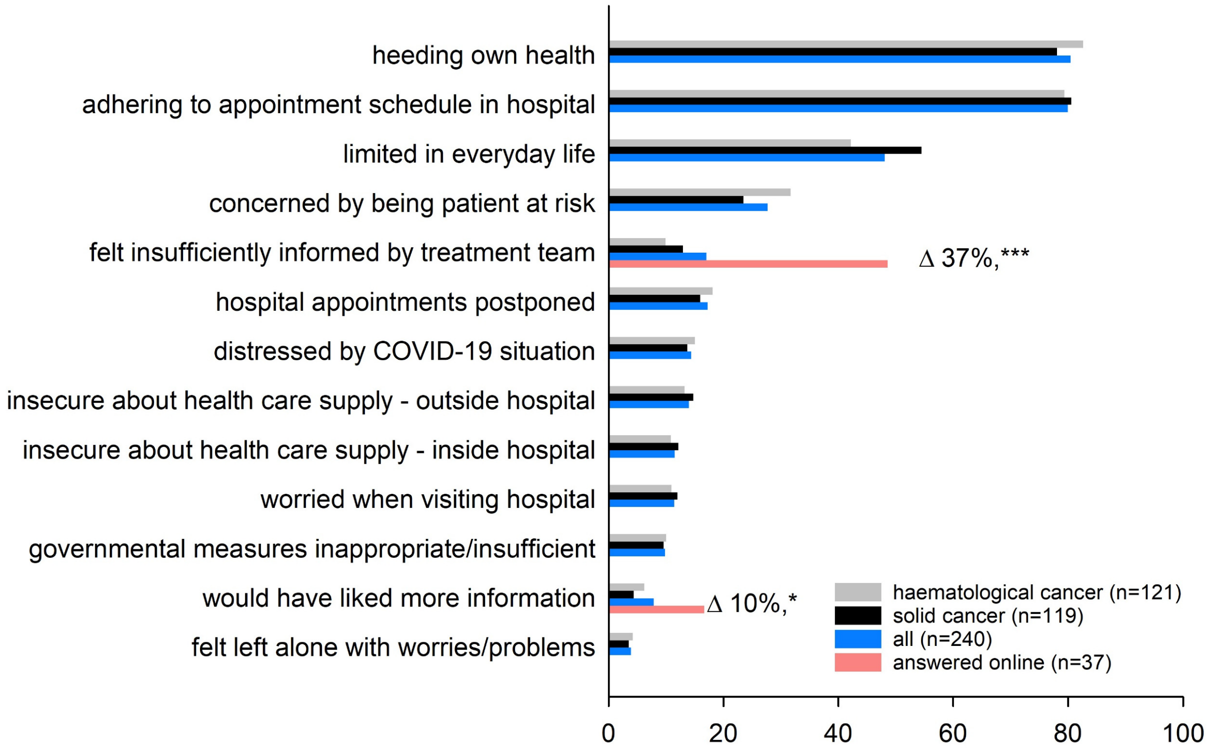


**Supporting Figure S3: Proportion of patients answering "very much" or "quite a bit" in the survey of COVID-19's impact on cancer patients' distress and everyday life stratified by patients with solid tumour *versus* haematological malignancies, and total cohort.** The answers do not significantly differ in relation to solid *versus* haematological cancer type. Note, that patients with solid tumours represent an overall younger cohort which is reflected in the more frequent limitations in everyday life (more distinct in Figure 3 with answerers stratified by age). Patients who answered the survey remotely (n=37; bright red line), felt significantly less informed by the treatment team and would have wanted more information. Δ = significant differences between categories, *** p<0.001, * p<0.01.

# Supporting Figures S4 – S5

### Longitudinal, intra-individual changes in HRQOL

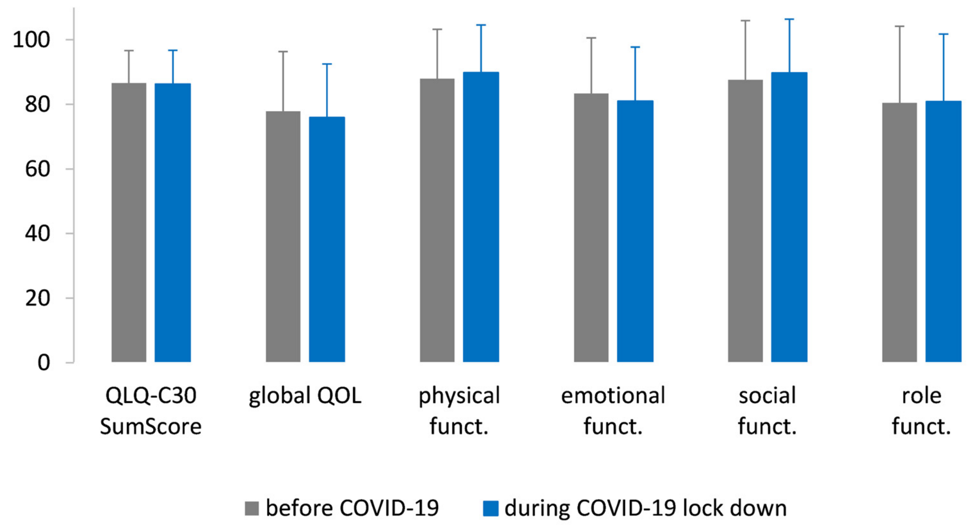


**Supporting Figure S4**: **Longitudinal changes in the mean of EORTC-QLQ-C30 summary score, global QOL and functions before and during the COVID-19 lockdown (n=47).** The changes are not significant. Comparing the means, all changes are below the threshold for minimally important differences (Cocks et al. 2012).

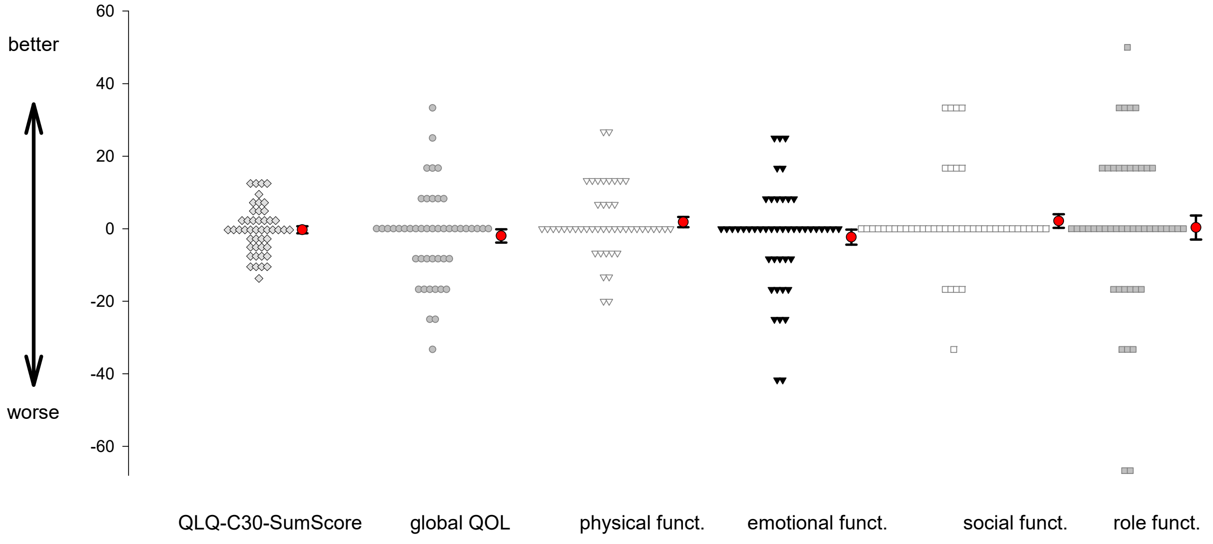


**Supporting Figure S5**: **Changes in EORTC-QLQ-C30 functions comparing answers per patient before and during the COVID-19 lockdown.** Within each dimension, the majority of the patients report stable conditions, except for summary score. No statistically significant trend towards deterioration or improvement could be detected. Still, individual patients reported strong changes (>20)(Osoba et al. 1998), especially for role functioning, but also for emotional and social functioning, and for global health, however, these changes cannot be separated from a regression to the mean effect. Red dot = mean plus standard error.

# Supporting Table S6 Odds Ratios

**Supporting Table S6: Association of five items assessed as impacted by COVID-19 in cancer patients’ everyday life and their association with female sex (*versus* male), younger age (*versus* patients older than 70 years), and impairments in EORTC-QLQ-C30** (summary score, global health/QOL, physical and emotional functioning) as odds ratios with 95% confidence intervals. Significant associations are highlighted in bold.

**Univariable Analyses**

| | **heeding own health** | | | | **adhering to appointment schedule** | | | | **limited in everyday life** | | | | **distressed by COVID19 situation** | | | | **concerned by being at-risk patient** | | | |
|---|---|---|---|---|---|---|---|---|---|---|---|---|---|---|---|---|---|---|---|---|
| | OR | LLC | ULC | p | OR | LLC | ULC | p | OR | LLC | ULC | p | OR | LLC | ULC | p | OR | LLC | ULC | p |
| female (*versus* male) | 1.38 | 0.71 | 2.67 | 0.343 | 0.62 | 0.30 | 1.27 | 0.194 | **1.70** | **1.01** | **2.86** | **0.047** | **2.85** | **1.32** | **6.17** | **0.008** | 0.93 | 0.52 | 1.65 | 0.803 |
| age | | | | 0.318 | | | | 0.357 | | | | **<0.001** | | | | 0.575 | | | | 0.171 |
| age 60-70 (*versus* >70) | 0.95 | 0.41 | 2.18 | 0.895 | 0.81 | 0.36 | 1.84 | 0.622 | **2.12** | **1.12** | **4.02** | **0.021** | 0.64 | 0.25 | 1.65 | 0.352 | 0.85 | 0.43 | 1.68 | 0.641 |
| age <60 (*versus* >70) | 0.60 | 0.28 | 1.28 | 0.186 | 0.52 | 0.21 | 1.27 | 0.151 | **3.74** | **1.94** | **7.24** | **<0.001** | 1.06 | 0.46 | 2.44 | 0.899 | 0.50 | 0.24 | 1.03 | 0.061 |
| QLQ-C30-SumScore < 87.9 | 0.83 | 0.42 | 1.66 | 0.603 | 0.98 | 0.47 | 2.03 | 0.957 | 1.42 | 0.83 | 2.45 | 0.205 | **4.75** | **1.60** | **14.1** | **0.005** | **3.10** | **1.53** | **6.26** | **0.002** |
| global QOL < 75.6 | 1.33 | 0.67 | 2.64 | 0.422 | 1.42 | 0.73 | 2.76 | 0.304 | 1.18 | 0.68 | 2.06 | 0.558 | **3.98** | **1.35** | **11.8** | **0.012** | 1.84 | 0.95 | 3.55 | 0.070 |
| physical funct. < 83 | 1.26 | 0.65 | 2.44 | 0.492 | 1.75 | 0.90 | 3.38 | 0.099 | 0.89 | 0.53 | 1.50 | 0.662 | **5.33** | **2.22** | **12.8** | **<0.001** | **2.27** | **1.26** | **4.09** | **0.006** |
| emotional funct. <71 | 1.08 | 0.55 | 2.14 | 0.824 | **2.15** | **1.03** | **4.50** | **0.041** | 1.53 | 0.89 | 2.64 | 0.122 | **5.30** | **2.39** | **11.8** | **<0.001** | **2.96** | **1.64** | **5.36** | **<0.001** |

**Multivariabel analyses**

| | **heeding own health** | | | | **adhering to appointment schedule** | | | | **limited in everyday life** | | | | **distressed by COVID19 situation** | | | | **concerned by being at-risk patient** | | | |
|---|---|---|---|---|---|---|---|---|---|---|---|---|---|---|---|---|---|---|---|---|
| | OR | LLC | ULC | p | OR | LLC | ULC | p | OR | LLC | ULC | p | OR | LLC | ULC | p | OR | LLC | ULC | p |
| female (*versus* male) | 1.49 | 0.74 | 3.01 | 0.264 | 1.56 | 0.77 | 3.15 | 3.006 | **1.32** | **0.75** | **2.33** | **0.334** | **2.47** | **1.04** | **5.85** | **0.040** | 0.81 | 0.43 | 1.53 | 0.516 |
| age | | | | 0.318 | | | | 0.254 | | | | **0.002** | | | | **0.800** | | | | 0.384 |
| age 60-70 (*versus* >70) | 0.97 | 0.41 | 2.32 | 0.950 | 1.04 | 0.48 | 2.24 | 0.930 | **2.05** | **1.04** | **4.02** | **0.038** | 1.08 | 0.37 | 3.15 | 0.891 | 1.03 | 0.49 | 2.17 | 0.944 |
| age <60 (*versus* >70) | 0.56 | 0.25 | 1.28 | 0.170 | 2.05 | 0.83 | 5.05 | 0.120 | **3.57** | **1.77** | **7.19** | **<0.001** | 1.39 | 0.52 | 3.75 | 0.515 | 0.60 | 0.27 | 1.34 | 0.215 |
| QLQ-C30-SumScore < 87.9 | 0.58 | 0.23 | 1.47 | 0.250 | 1.71 | 0.71 | 4.15 | 0.235 | 1.88 | 0.87 | 4.09 | 0.109 | 1.26 | 0.31 | 5.16 | 0.749 | 2.26 | 0.91 | 5.63 | 0.079 |
| global QOL < 75.6 | 1.47 | 0.64 | 3.38 | 0.363 | 0.87 | 0.39 | 1.94 | 0.735 | 1.09 | 0.54 | 2.18 | 0.808 | 1.30 | 0.37 | 4.60 | 0.686 | 0.85 | 0.38 | 1.91 | 0.689 |
| physical funct. < 83 | 1.23 | 0.52 | 2.88 | 0.635 | 1.24 | 0.51 | 3.00 | 0.640 | 0.64 | 0.31 | 1.34 | 0.238 | 2.89 | 0.93 | 8.97 | 0.067 | 1.02 | 0.47 | 2.23 | 0.953 |
| emotional funct. <71 | 1.01 | 0.45 | 2.27 | 0.987 | 1.45 | 0.62 | 3.42 | 0.391 | 1.24 | 0.63 | 2.45 | 0.529 | **3.17** | **1.21** | **8.28** | **0.019** | **2.21** | **1.09** | **4.49** | **0.029** |
| ominbus test | | | | 0.676 | | | | 0.105 | | | | **0.002** | | | | **<0.001** | | | | **0.010** |

OR= odds ratio, 95% conficence limits: LLC=lower limit of confidence, ULC=upper limit of confidence
patients restricted in global QOL or EORTC-QLQ-C30 summary score relative to general population normative data for Austria (Nolte, Liegl et al. 2019)
patients restricted in physical and emotional functioning relative to thresholds of clinical importance according to Giesinger, Loth et al. 2020

## References Supporting Information

Cocks K, King MT, Velikova G, et al. Evidence-based guidelines for interpreting change scores for the European Organisation for the Research and Treatment of Cancer Quality of Life Questionnaire Core 30. European Journal of Cancer 2012; 48(11): 1713-21.

Giesinger JM, Loth FLC, Aaronson NK, et al. Thresholds for clinical importance were established to improve interpretation of the EORTC QLQ-C30 in clinical practice and research. J Clin Epidemiol 2020; 118: 1-8.

Nolte S, Liegl G, Petersen MA, et al. General population normative data for the EORTC QLQ-C30 health-related quality of life questionnaire based on 15,386 persons across 13 European countries, Canada and the Unites States. European journal of cancer (Oxford, England: 1990) 2019; 107: 153-63.

Osoba D, Rodrigues G, Myles J, Zee B, Pater J. Interpreting the significance of changes in health-related quality-of-life scores. Journal of clinical oncology: official journal of the American Society of Clinical Oncology 1998; 16(1): 139-44.
